# Evolutionarily stable strategy analysis and its links to demography and genetics through invasion fitness

**DOI:** 10.1098/rstb.2021.0496

**Published:** 2023-05-08

**Authors:** Jeremy Van Cleve

**Affiliations:** Department of Biology, University of Kentucky, Lexington, KY 40506 USA

**Keywords:** evolutionarily stable strategy, lineage fitness, kin selection, group selection, reduction principle, variable environments

## Abstract

Evolutionarily stable strategy (ESS) analysis pioneered by Maynard Smith and Price took off in part because it often does not require explicit assumptions about the genetics and demography of a population in contrast to population genetic models. Though this simplicity is useful, it obscures the degree to which ESS analysis applies to populations with more realistic genetics and demography: for example, how does ESS analysis handle complexities such as kin selection, group selection and variable environments when phenotypes are affected by multiple genes? In this paper, I review the history of the ESS concept and show how early uncertainty about the method lead to important mathematical theory linking ESS analysis to general population genetic models. I use this theory to emphasize the link between ESS analysis and the concept of *invasion fitness*. I give examples of how invasion fitness can measure kin selection, group selection and the evolution of linked modifier genes in response to variable environments. The ESSs in these examples depend crucially on demographic and genetic parameters, which highlights how ESS analysis will continue to be an important tool in understanding evolutionary patterns as new models address the increasing abundance of genetic and long-term demographic data in natural populations.

This article is part of the theme issue ‘Half a century of evolutionary games: a synthesis of theory, application and future directions’.

## 1. Introduction

Although Richard Lewontin was the first to introduce game theory into biology in 1961 [1], very few papers on the topic of game theory and biology were published in the following decade. It was not until 1973 when John Maynard Smith and George Price introduced the evolutionarily stable strategy (ESS) and applied it to the study of animal behaviour [2] that biologists more widely came to appreciate the relevance and use of game theoretic concepts and tools for questions in evolution biology and ecology. Specifically, Maynard Smith and Price posited that individual fitness could be viewed as an analogue of the game-theoretic notion of *utility* or *pay-off*, which is the quantity that measures what agents optimize in pursuit of their objectives [3]. Viewed this way, the ESS is a refinement of the famous Nash equilibrium [4]. Maynard Smith explained the ESS concept in more detail in an important paper in 1974 [5], introduced the famous *Hawk-Dove* game in his study of asymmetric games in 1976 [6], and summarized the nascent field of evolutionary game theory in his now classic 1982 book [7]. In the 50 years since the 1973 paper, evolutionary game theory has become an essential tool in evolutionary and behavioural ecology and includes rich theoretical work that delves into foundational evolutionary and game-theoretic concepts and mathematical and simulation models that predict ESSs for many specific biological systems. Evolutionary game theory has also strongly influenced the social sciences including, economics, political science, psychology and anthropology and has drawn applied mathematicians, computer scientists and physicists into mathematical biology.

The role of evolutionary game theory and the ESS method specifically in evolutionary biology has been at the crux of some of the most important conceptual debates in the field including the role of natural selection and adaptation *vis-a-vis* other evolutionary forces (e.g. [8–14]) and the importance of kin and group selection relative to individual selection (e.g. [15–28]). These debates might be captured in part by the following two questions: (i) how do ESS models that focus on individual fitness capture the effects of kin selection or group selection? and (ii) how do ESS models account for recombination, mutation, migration and other evolutionary forces? In this work, I will describe how conceptual advances in evolutionary theory since Maynard Smith & Price [2] have shed light on both of these questions and have led to a broader understanding of the ESS method and a more integrative framework for understanding how multiple evolutionary forces in complex populations can lead to diverse phenotypes.

Question (i) derives in part from how Maynard Smith introduced his work on ESSs; he argued that an ESS could provide an explanation for the evolution of behaviours where ‘selection acts entirely at the individual level, but in which the success of any particular strategy depends on what strategies are adopted by other members of the population’ [5, p. 210] and is not ‘due to group or species selection’ [2, p. 15] or selection on ‘close relatives’ ([5], p. 210) as might be the case for kin selection [29]. In setting up this dichotomy, Maynard Smith implied that the ESS method may not apply when kin or group selection are involved. As I will describe below, evolutionary theorists have since realized (including Maynard Smith himself [8, p. 33]) that the issue of the applicability of the ESS method is really orthogonal to issues of individual versus group versus kin selection and instead captures in what sense evolution via natural selection optimizes fitness given a specific measure of fitness. Issues of individual versus group versus kin selection are about the *units* or *levels* at which selection acts and how fitness should be measured to account for selection at those levels. Lewontin also hints at the levels of selection question in his 1961 game theory paper when he discusses the relative merits of individual-level measures of fitness like intrinsic or Malthusian growth rate *r* versus population-level measures like mean fitness $\bar{w}$ [1, pp. 400–401] as analogues of utility. The levels of selection question became a major topic of study in evolutionary theory (e.g. [30–37]) and philosophy of biology [38–45] and continues to generate substantial research (e.g. [46–48]). For the purpose of explaining how an ESS can capture selection at multiple levels and among relatives or kin, I will argue below that the right measure of utility is the *invasion fitness* [49,50] or *lineage fitness* [51–53] of a mutant allele at a single genetic locus, which can be shown to be functionally equivalent to a measure of inclusive fitness [53,54], which is a concept originally proposed by W. D. Hamilton in relation to kin selection [29].

The origin of question (ii) rests in a different set of debates in evolutionary biology regarding the relative role of natural selection *vis-a-vis* other evolutionary forces, such as mutation, recombination and migration, in explaining organismal phenotypes. Early in the twentieth century, R. A. Fisher’s ‘fundamental theorem of natural selection’ (FTNS) [55] established a mathematical expression of the importance of natural selection. The FTNS states that the increase in the mean fitness of a population is equal to the genetic variance in fitness and thus seems to imply that populations always become better adapted to their environments (since genetic variance is always non-negative) and even that fitness is maximized over the long term. It is hard to overstate the importance of the FTNS in shaping the direction of evolutionary theory. The FTNS came to typify the idea that evolutionary change is dominated by natural selection as a fitness optimizing force. Hamilton appealed to this idea in his original paper on kin selection [29] and theorized that inclusive fitness is the fitness quantity that is maximized.

Subsequent work by population geneticists revealed the weakness of this understanding of the FTNS by showing that mean fitness can decrease owing to frequency-dependent selection [30,56] or recombination among multiple genetic loci [57–60]. Further, studies in the 1960s of the rates of molecular evolution [61,62] and levels of polymorphism [63,64] in a number of species spurred the development of the neutral [65,66] and nearly neutral [67,68] theories [69] of molecular evolution. These theories posited that many mutations are only weakly affected by natural selection, and thus their fate is governed mostly by genetic drift [66]. Consequently, by the 1970s, evolutionary biologists were heavily debating the relative role of natural selection versus other forces in shaping evolutionary patterns [70,71], and some biologists including Lewontin specifically questioned the importance of fitness maximization [9,59]. Maynard Smith was a clear proponent of fitness maximization and viewed it natural to ‘[assume] that evolution has occurred by natural selection’ [8, p. 31]. He proposed the ESS method developed by Price and himself as the appropriate tool for predicting phenotypic evolution and particularly for social traits. While Maynard Smith acknowledged that ESS models make a number of biological assumptions including that traits have a simple genetic basis and that appropriate genetic variation exists [8], he believed that the limitations imposed by these assumptions are biologically reasonable. This view that simplifying away genetic complexities or constraints is generally reasonable was termed the ‘phenotypic gambit’ by Grafen [72] and came to dominate evolutionary theory in behavioural and evolutionary ecology [73]. The near-singular focus of evolutionary game theory and the ESS method on natural selection has become less defensible given the recent and increasing abundance of genomic and transcriptomic data for a diverse set of species (e.g. [74–78]); specifically, some have argued that understanding these data requires moving beyond the gambit with more explicit consideration of complex genetic and demographic mechanisms (e.g. [79–82]). Question (ii) arises here and asks whether the ESS method can be modified to accommodate other evolutionary forces such as mutation and recombination. I will argue below that the ESS method is more general than originally imagined by Maynard Smith & Price [2] in that it can in fact be incorporated into an evolutionary framework where natural selection and other evolutionary forces combine to shape the short- and long-term evolution of traits. Though this framework is quite general, it does have assumptions and limitations that are discussed in §§8 and 9.

## 2. What is an evolutionarily stable strategy?

The definition of an ESS is relatively simple and only involves a set of phenotypes, a measure of fitness (the evolutionary measure of pay-off), and a stability condition. Suppose that two individuals interact and the phenotypes that characterize their behaviour in the interactions are measured with the variables *x* and *y*. The phenotypes can be drawn from a set of discrete or continuous values that represents a single trait like body size or propensity to cooperate. For the purpose of explaining the theory more concretely, assume that individuals can choose one of two possible behaviours in the interaction, *C* and *D*. The phenotypes *x* and *y* measure the probability that individuals in an interaction with those phenotypes choose behaviour *C*, and 1 − *x* and 1 − *y* are the probabilities those individuals choose behaviour *D* instead (probabilistic phenotypes are called *mixed strategies* in game theory [83]). For example, the behaviour *C* could be cooperation in the interaction and *D* could be a lack of cooperation. Let *w*(*x*, *y*) be the fitness that an individual with phenotype or strategy *x* receives when interacting with an individual with phenotype or strategy *y*. Fitness *w* for the moment is simply the survival rate (i.e. viability selection) but as we will see we can define much more general fitness measures. The stability condition [2,5] says that a phenotype x* is an ESS, or is evolutionarily stable (ES), when one of two scenarios occurs. For any alternative strategy *y*, either *y* receives less fitness when interacting with x* than x* receives when interacting with itself:

2.1a $$w(x^{*},x^{*})>w(y,x^{*}),$$

or *y* gets the same fitness interacting with x* as x* does with itself and x* receives more fitness interacting with *y* than *y* does with itself:

2.1b $$w(x^{*},y)>w(y,y)\;\text{when}\;w(x^{*},x^{*})=w(y,x^{*}).$$

The two conditions in equations (2.1) are together equivalent to requiring that phenotype x* receives higher average fitness than any alternative phenotype *y* when *y* is rare in the population [5,84]. The phenotype x* is called a *strict* ESS when condition (2.1*a*) holds and a *weak* ESS when condition (2.1*b*) holds.

To make the ESS method more concrete, consider a scenario where *C* and *D* correspond to cooperating and doing nothing, respectively. We might start with the two deterministic or *pure strategies*: *x* = 1 individuals always cooperate and *x* = 0 individuals never cooperate. There are four possible interaction pairs and their pay-offs can be collected into the pay-off matrix:

2.2 $$W=\left(\begin{array}{cc} w(1,1) & w(1,0)\\ w(0,1) & w(0,0) \end{array}\right).$$

Individuals benefit from having a partner who cooperates, which translates into *w*(1, 1) > *w*(1, 0) and *w*(0, 1) > *w*(0, 0). Since cooperation is personally costly, an individual has less pay-off when it cooperates than when it does not given its partner also does not cooperate, or *w*(1, 0) < *w*(0, 0). Moreover, that cost is pervasive enough so that cooperation is more costly than non-cooperation even when the partner does cooperate, or *w*(1, 1) < *w*(0, 1). Finally, two individuals that cooperate do better than two individuals that do not, or *w*(1, 1) > *w*(0, 0). These pay-off inequalities can be summarized as *w*(0, 1) > *w*(1, 1) > *w*(0, 0) > *w*(1, 0) and they characterize the famous Prisoner’s Dilemma game [85], which often involves public goods. Applying the ESS conditions, equation (2.1) shows that noncooperation is the ESS or x* = 0 for the prisoner’s dilemma. For an example of a mixed-strategy ESS where 0 < x* < 1, assume that the pay-offs obey the conditions *w*(0, 1) > *w*(1, 1) > *w*(1, 0) > *w*(0, 0), which characterize a Hawk-Dove [2,6] or Snowdrift game [86]. The only change from the Prisoner’s Dilemma conditions is the reversal of the last inequality so that now *w*(1, 0) > *w*(0, 0). This inequality indicates that cooperation in the Hawk-Dove game yields enough pay-off to the cooperator so that both individuals prefer that at least one of them cooperates. A Hawk-Dove game might describe two individuals foraging for resources where its beneficial for at least one individual to expend the effort to search. Applying the ESS conditions in equations (2.1) reveals that neither pure strategy *x* = 1 nor *x* = 0 is an ESS. Rather, the Hawk-Dove game has a mixed strategy ESS where individuals cooperate with probability x* = (*w*(1, 0) − *w*(0, 0))/(*w*(1, 0) − *w*(0, 0) + *w*(0, 1) − *w*(1, 1)).

## 3. How is an evolutionarily stable strategy related to population genetic dynamics?

The ESS conditions in equations (2.1) do not reference the genetic basis of the trait *x* nor do they describe how the frequency or mean value of the trait changes over time. Population genetics models, on the other hand, are built to measure the dynamical process of evolution via the combined effects of natural selection and segregation, recombination, mutation, genetic drift and other evolutionary forces [87,88]. A natural question then is how the long-run behaviour of a population genetic model (e.g. its equilibrium genotypes and induced phenotypes) compares to the equilibrium phenotypes obtained from an analogous ESS model. This question was tackled by mathematical population biologists beginning in the late 1970s and through 1980s and 1990s (e.g. [89–101]).

Early mathematical analyses began by using the simplest population genetic model, which is one that assumes a *haploid asexual* population with two genotypes. Assume the two genotypes correspond to the phenotypes *x* = 1 that chooses *C* with certainty and *x* = 0 that chooses *D* with certainty, respectively. The fitness of each of the four interaction pairs is given in the pay-off matrix *W* in equation (2.2). Suppose that *p* measures the frequency of the phenotype *x* = 1 that always chooses *C*. Pairs are assumed to form randomly according to the frequencies *p* and 1 − *p* of the two types. The equilibria of this model are either fixation of one of the two genotypes resulting in a population of individuals that always chooses *C*, *p* = 1, or always chooses *D*, *p* = 0 or a polymorphism of both genotypes, 0 < *p* < 1, resulting in a population where some choose *C* and some choose *D*. The analogous ESS model of this scenario applies condition (2.1) to the four possible fitness values in the pay-off matrix *W* in equation (2.2) and determines which phenotype, *x* = 1 or *x* = 0, is ESS or whether a mixed strategy (0 < *x* < 1) is ESS. These early analyses showed that the values of *x* that are an ESS are also values of *p* that produce stable equilibria in the haploid asexual population genetic model [89–91,102].

This correspondence between the population genetic and ESS models is not particularly surprising since natural selection is the only evolutionary force acting in the haploid asexual model. However, a similar correspondence between ESSs and population genetic models also applies to a *diploid sexual* population reproducing in discrete time where Mendelian segregation breaks up diploid genotypes every generation [92,96]. For this scenario, the phenotype is controlled by a single genetic locus with *a* possible alleles *A*_1_, …, *A*_*a*_. Diploid genotype *A*_*i*_ *A*_*j*_ chooses behaviour *C* with probability *x*_*ij*_ and behaviour *D* with probability 1 − *x*_*ij*_. If there are no restrictions on the *x*_*ij*_, then dominance is possible between the alleles. Like the asexual haploid model, diploid individuals interact in pairs according to their probability in the population and mating is random. Let **p** = (*p*_1_, …, *p*_*a*_) be the vector of allele frequencies for alleles *A*_1_, …, *A*_*a*_ in the parental generation. The mean phenotype or probability of choosing *C* in the population after reproduction is then

$$x=x(p)=\sum_{i,j}p_{i}p_{\;j}x_{ij}.$$

Eshel & Lessard showed [92,96] that ESSs x* of the pay-off matrix *W* are stably maintained in the diploid population model when those ESSs are produced by some vector of allele frequencies p* such that x* = *x*(p*). This correspondence between ESSs and population genetic equilibria also holds for continuous-time diploid models [97,98,102,103] and for models with multiple loci when the probability of a genotype choosing a phenotype is an additive function of effects from each locus (i.e. additive non-epistasis) [92].

## 4. Evolutionarily stable strategies and long-term evolution

An important limitation of the above results connecting ESS phenotypes x* to equilibria in diploid single and multilocus population genetic models is that the genotype–phenotype map may not produce phenotypes that are ESS and thus those ESS phenotypes are unattainable in the short-term. Specifically, the *x*_*ij*_ may have values so that no population composition **p** of alleles *A*_*i*_ can produce the ESS phenotype x*. Moreover, as mentioned above, multilocus systems more generally (i.e. systems with non-additive interactions or epistasis) do not guarantee that genotypes with higher marginal fitness^1^ increase in frequency, which is a requirement for the correspondence between ESS and population genetic equilibria described above [92,102]. Thus, the genetic system may impose constraints on either the range of possible phenotypes or on the ability of selection to increase the frequency of high marginal fitness genotypes.

Intriguingly, Eshel, Feldman and others discovered that these constraints might not be so important on longer-term evolutionary timescales. The important idea is that the conditions under which a genetic equilibrium is stable given a fixed set of alleles or genotypes does not determine whether a new and rare allele or genotype outside of that fixed set might invade the population. When an equilibrium is stable given a fixed set of alleles or genotypes, it is called *internally stable* [99]. If that equilibrium also resists invasion by new alleles or genotypes outside the fixed set, it is called *externally stable* [99,104]. Equilibria that are internally stable may not be externally stable. For example, suppose there is heterozygote advantage in a population with two alleles at a single diploid locus. The internally stable equilibrium is a polymorphism where both alleles are present in positive frequency. However, a new allele can invade the population if it generates the original heterozygote fitness in a heterozygote with either original allele and in a homozygote with itself. Hence, the internally stable polymorphism is not externally stable. The invasion of this new allele will lead to its fixation, which is a new internally stable equilibrium that may or may not be externally stable to further invasions depending on the genotype–phenotype map (i.e. how the genotypes *A*_*i*_ *A*_*j*_ generate fitness values *w*_*ij*_). Given a fixed set of genotypes, the evolution of those genotypes to an internally stable equilibrium is called *short-term evolution,* whereas the process of new mutant invasions that move the population from one internally stable equilibrium to another is called *long-term evolution* [99–101,105–108] and eventually leads to an externally stable equilibrium. The process of long-term evolution assumes that mutations are infrequent enough so that a new mutation has sufficient time to either go extinct or reach a new short-term equilibrium before additional mutations occur. Exactly how infrequent this is depends on additional parameters such as the strength of selection and the population size and requires analysing the stochastic interaction between mutation, drift and selection [109–112]. Long-term evolution involving sequential substitution of genotypes is sometimes called the ‘streetcar process’ [100] or the ‘trait substitution sequence’ [49,113,114] and is the fundamental evolutionary model of the *adaptive dynamics* approach [115–117] described in §9.

Remarkably, despite the additional complexity of the long-term process, the equilibria of the long-term evolution process, not the short-term process, are what most closely align to what is predicted by the ESS criteria in equations (2.1). Eshel, Feldman, Lessard and others demonstrated that externally stable equilibria are those that generate phenotypes x* that are ESS [95,99–101,104,106,118]. Specifically, suppose that the probability of selecting between behaviours *C* and *D* is determined by two loci *A* and *B* with alleles *A*_1_, …, *A*_*a*_ and *B*_1_, …, *B*_*b*_, respectively, and with recombination rate 0 < *R* ≤ 1/2 between the loci. The vector **p** = (…, *p*_*ij*_, …) tracks the frequencies of the chromosomes (e.g. *A*_*i*_ *B*_*j*_) after selection and recombination. Individuals with diploid genotype *A*_*i*_*B*_*j*_/*A*_*k*_*B*_*l*_ chooses behaviour *C* with probability *x*_*ijkl*_ and behaviour *D* with probability 1 − *x*_*ijkl*_. The mean frequency of phenotype *x* in the population after reproduction is

$$x=x(p)=\sum_{i,j,k,l}p_{ij}p_{kl}x_{ijkl}.$$

If we assume that the fitness function *w*(*x*, *y*) is linear in the phenotypes *x* and *y* (which is a reasonable assumption given the phenotypes are probabilities of selecting among behaviours *C* and *D*), then the mean or marginal fitness of an individual with genotype *A*_*i*_*B*_*j*_/*A*_*k*_*B*_*l*_ interacting with other individuals in the population according to their frequencies is *w*(*x*_*ijkl*_, *x*). Likewise, the mean fitness in the population is then

$$\bar{w}=\sum_{i,j,k,l}w(x_{ijkl},x)p_{ij}p_{kl}=w(x,x).$$

Now, assume that a new mutant allele *A*_*μ*_ (increasing the total number of alleles at locus *A* from *a* to *a* + 1) arrives at low frequency in a population at an internally stable equilibrium $\hat{p}$ with *resident* or wild-type mean phenotype $\hat{x}=x(\hat{p})$. An individual with this new allele chooses behaviour *C* with probability *x*_*μjkl*_ if its genotype is *A*_*μ*_*B*_*j*_/*A*_*k*_*B*_*l*_ and behaviour *D* with probability 1 − *x*_*μjkl*_. A linear stability analysis shows that this allele will either initially increase or decrease in frequency at a geometric rate *λ* that is the magnitude of the largest eigenvalue of a Jacobian matrix [119]. The eigenvalue *λ* has an associated normalized eigenvector **u** whose elements *u*_*j*_ measure the frequency of mutant chromosomes (i.e. chromosomes *A*_*μ*_ *B*_*j*_) once the invasion process has stabilized (i.e. has reached stationarity [120]). Eshel & Feldman [95,106] showed that this geometric growth rate is

4.1 $$\lambda =\frac{w(x_{\mu },\hat{x})}{w(\hat{x},\hat{x})},$$

where *λ* > 0 and

4.2 $$x_{\mu }=\sum_{\;j,k,l}u_{\;j}{\hat{\;p}}_{kl}x_{\mu jkl},$$

is the average probability of choosing behaviour *C* among mutant individuals once the invasion process has stabilized. Note that since the mutant allele is rare, mutant individuals can have only one mutant chromosome; the other chromosome has no mutant alleles at locus *A* and remains at the internal equilibrium frequency ${\hat{\;p}}_{kl}$. A sufficient condition for the equilibrium with phenotype $\hat{x}$ to be externally stable is that *λ* < 1 or from equation (4.1):

$$w(x_{\mu },\hat{x})<w(\hat{x},\hat{x}),$$

for all possible mutants *A*_*μ*_ that generate mutant phenotype *x*_*μ*_. This condition is in fact the condition for a strict ESS from equation (2.1*a*) and can be used to prove the following result for large, randomly mating, diploid populations with two-locus genetic systems [95,99,106].

**Result 4.1.**

Any phenotype $\hat{x}$ generated by an externally stable equilibrium is an ESS. Moreover, if an ESS x* is generated by an internally stable equilibrium $\hat{p}$, $\hat{p}$ is externally stable.

Work by Liberman [104] on more than two loci probably extends Result 4.1 to any finite number of loci. Result 4.1 shows that ESSs determined by the condition (2.1) of Maynard Smith & Price [2,5] are stable states of the long-term evolutionary process. Assuming that long-term evolution is a reasonable model of phenotypic evolution, Result 4.1 demonstrates the importance of natural selection on phenotypes in determining the course of phenotypic evolution and provides a robust mathematical justification for the use of the ESS method in building phenotypic evolution models.

## 5. Invasion fitness and evolutionarily stable strategies

While Result 4.1 emphasizes the role of the ESS and natural selection on phenotypes in phenotypic evolution, the external stability approach used to prove Result 4.1 reveals a more general viewpoint where ESSs can depend in important ways on the genetic and demographic parameters of a population. External stability analysis assumes that the population is at some genetic equilibrium where new mutant alleles can invade at low frequency and looks for a genetic equilibrium and its resulting phenotype that resists invasion by any mutant allele (within a set of possible phenotypic effects). Before the mathematical machinery of external stability was developed, Hamilton appreciated the conceptual importance of uninvadable phenotypes in his study of the sex ratio, and he called sex ratios that resisted invasion by any alternative sex ratio *unbeatable* phenotypes [121]. As we saw above, determining whether a phenotype can be unbeatable and its genetic equilibria externally stable involves calculating the magnitude of the eigenvalue *λ* associated with the linear dynamical system that approximates the dynamics of the mutant allele frequency when rare. The magnitude of the eigenvalue *λ* measures the geometric growth rate (e.g. equation (4.1)) of all mutant genotypes in a population of resident genotypes. If there are multiple possible genotypes with the mutant allele, as is the case for the two-locus model in §4 where there may be many possible alleles at the non-mutant locus, then mutant genotype frequencies during the invasion process are given by the normalized right eigenvector **u** of the linear system. Thus, *λ* really measures the fitness of the whole mutant genetic lineage that originates from the original invading mutants. The growth rate *λ* is often called the *invasion fitness* [49,50]^2^ and sometimes called the *lineage fitness* [51–54]. In effect, the invasion or lineage fitness measures the average number of offspring copies produced by a mutant allele sampled randomly from among all the genotypes in the mutant lineage [53]. Assuming that *λ* > 0, the phenotype x* is unbeatable whenever

5.1 $$\lambda (y,x^{*})<1\;\text{for all}\;y\neq x.$$

Regardless of the complexity of the genetics, demography or ecology of a population, we can in principle perform an external stability analysis^3^ and use invasion fitness to determine whether there are unbeatable phenotypes.

It should already be evident given Result 4.1 that the unbeatable phenotypes obtained via external stability are closely connected to phenotypes obtained via an ESS analysis using condition (2.1). In the general case for an arbitrary genetic and demographic scenario, we assume only that we have the invasion fitness *λ*(*y*, x*) of a rare mutant *y* in the population that otherwise has mean phenotype x*. Starting with the condition for a strict ESS, *w*(x*, x*) > *w*(*y*, x*) for *y* ≠ x* from equation (2.1*a*), we can convert that condition to one in terms of *relative fitness* by dividing by the mean population fitness *w*(x*, x*) to obtain

5.2 $$\frac{w(y,x^{*})}{w(x^{*},x^{*})}=\omega (y,x^{*})<1\;\text{for all}\;y\neq x^{*},$$

where *ω*(*y*, x*) is the relative fitness of a rare mutant allele with phenotype *y* in a population with mean phenotype x*. The strict ESS condition in equation (5.2) using relative fitness is identical to the condition for uninvadability in condition (5.1) using invasion fitness, which means that unbeatable or uninvadable phenotypes are strict ESSs.^4^

Since we can in principle calculate invasion fitness for populations with complex genetics and demography, we can obtain strict ESSs in those populations even though Result 4.1 may not apply. However, those ESSs will almost certainly depend on genetic and demographic parameters of the population as the invasion fitness captures the effect of genetic and demographic processes on the growth of invading mutant lineages.^5^ For example, the ESS for the sex ratio of a population depends on the genetic structure of the population: in a large, randomly mating population, the sex ratio is 1:1 when the ratio is determined by a diploid autosomal gene [121,123,124], but it can take other values when the gene is sex-linked or the genetic system is more complex like in insects that are haplodiploid [121,125]. The fact that ESSs can incorporate genetic and demographic forces alongside natural selection shows that the ESS method is a more general tool than potentially originally envisioned by Maynard Smith and other proponents of the phentoypic gambit. Below, we will describe two cases that capture this flexibility: group- and class-structured populations with kin selection and populations in variable environments with variable phenotypes.

## 6. Inclusive fitness, group-structured populations and evolutionarily stable strategies

In a populations where individuals live in groups and migration (or dispersal) is limited, individuals are more genetically related to their groupmates than to individuals in different groups [126–128], which means there is a possibility for kin selection to drive the evolution of prosocial phenotypes [29,129,130]. Moreover, group structure allows for competition among groups for resources and the possibility of selection among groups favouring more prosocial groups given sufficient group integrity [131–135]. In fact, the Price equation [17,136] can be used to show that kin and group selection perspectives on fitness both lead to the same predictions when applied to the same population model [20,137–141]. Thus, we should expect that predicting an ESS for prosocial behaviour in a group structured population by calculating invasion fitness will capture the effects of both kin and group selection. To see this, we will outline the invasion fitness analysis of this scenario performed by Lehmann and colleagues [53,54]. This analysis also provides a formal justification for the intuition of Maynard Smith [8] and others that a correct fitness measure for ESS analysis is Hamilton’s inclusive fitness [29] by showing that inclusive fitness is monotonically related to invasion fitness.

Suppose that a population is composed of an infinite number of groups each containing *n* individuals; groups can be thought as demes or patches each equally connected to one another at some rate of migration (i.e. Wright’s island model [142]). Each individual can belong to one of *c* demographic classes, such as age, sex or caste (e.g. as workers and queens in social insects). Phenotypes are produced by alleles at a single haploid genetic locus. Alleles can be pleiotropic and code for a specific phenotype for each class: phenotype **x** = (*x*_1_, …, *x*_*c*_) where *x*_*s*_ is the phenotype for class *s*. We assume that once at a genetic and demographic equilibrium with phenotype $\hat{x}$, the population stabilizes at *n*_*s*_ individuals in class *s* in each group where the group size is just the sum of all the individuals in each class or $n=\sum_{s}n_{s}$.

We introduce a mutant phenotype **y** at low frequency into a population where all individuals have the resident phenotype ◯. One way to track the number of mutant individuals in the whole population is to track the number of groups with *k* mutants. Since each individual belongs to a specific class, we actually track how many mutant individuals *k*_*s*_ are in each class *s* in a group or **k** = (*k*_1_, …, *k*_*c*_) where $k=\sum_{s}k_{s}$. The number of possible states for **k** increases quickly with the group size *n* and the number of classes *c* but is finite. For simplicity of notation, we assume there is an ordering of these states so that we can use **k** to index vectors and matrices below. When the mutant phenotype is rare, growth of the mutant population can be modelled using a matrix population model [120] or multitype branching process [143] with a projection matrix **A**(**y**, $\hat{x}$) = [*a*_**k**′**k**_(**y**, $\hat{x}$)] where the *a*_**k**′**k**_(**y**, $\hat{x}$) element measures the expected number of groups with **k**′ mutants with phenotype **y** that result from the reproduction occurring in a group with **k** mutants in a population resident for phenotype $\hat{x}$. The largest eigenvalue *λ*(**y**, $\hat{x}$) of the projection matrix **A**(**y**, $\hat{x}$) is the invasion or lineage fitness in this population and satisfies

6.1 $$A(y,\hat{x})u(y,\hat{x})=\lambda (y,\hat{x})u(y,\hat{x}),$$

where the associated eigenvector **u**(**y**, $\hat{x}$) has elements *u*_**k**_ that measure the relative frequency of groups with **k** mutants when the invasion process has stabilized. The elements *a*_**k**′**k**_(**y**, $\hat{x}$) of the projection matrix can be rewritten in terms of an individual relative fitness function *ω*_*s*′*s***k**_(**y**, $\hat{x}$), which measures the number of class *s*′ mutant offspring in the population in the next generation produced by a class *s* mutant individual in a group with **k** mutants. Relative fitness is the right measure here since we measure offspring production after migration, density-dependent survival and all other life-cycle events. The projection matrix and fitness function are then related through the expression for the total number of class *s*′ offspring produced by mutant individuals in a group with **k** mutants:

6.2 $$\sum_{k^{\prime }}k_{s^{\prime }}^{\prime }a_{k^{\prime }k}(y,\hat{x})=\sum_{s}k_{s}\omega_{s^{\prime }sk}(y,\hat{x}).$$

Lehmann *et al.* [53,54] use equation (6.2) to show that the invasion or lineage fitness of mutant phenotype **y** in a resident population with phenotype $\hat{x}$ is given by

6.3 $$\lambda (y,\hat{x})=\sum_{k,s^{\prime },s}\omega_{s^{\prime }sk}(y,\hat{x})q_{k,s}(y,\hat{x}),$$

where *q*_**k**,*s*_(**y**, ◯) is the probability (when the invasion process has stabilized) a randomly sampled mutant allele from the mutant lineage is in a class *s* individual in a group with **k** mutants and is given by

$$q_{k,s}(y,\hat{x})=\frac{k_{s}u_{k}(y,\hat{x})}{\sum_{k,s}k_{s}u_{k}(y,\hat{x})}.$$

The invasion fitness expression in equation (6.3) for a group- and class-structured population is conceptually identical to the one for a single population with two loci in equation (4.1): both expressions show that the mutant successfully invades when mutant alleles more than replace themselves when averaged across all the genetic contexts (i.e. possible alleles at other loci) and demographic contexts (i.e. possible group compositions in terms of mutant allele frequency in each class) in which the mutant lineage occurs during the invasion process.

The invasion fitness in equation (6.3) can also be expressed in terms of inclusive fitness in order to isolate the effect on fitness of mutant allele expression in the focal individual (direct effect) and in genetically related individuals (indirect effect). There are some important technical subtleties as to how this can be accomplished that we will leave out to keep the presentation simple (see ‘Supplement B’ in [54], for details). The essence of the method is the same as the one found in many analyses that derive inclusive fitness using the Price equation (e.g. [20,137,144,145]): the fitness of a focal individual, here *ω*_*s*′*s***k**_(**y**, $\hat{x}$), is decomposed into cost and benefit terms reflecting the marginal effects on fitness^6^ of expressing the mutant phenotype *y*_*s*_ relative to the resident phenotype ${\hat{x}}_{s}$. The cost term *c*_*s*′*s*_(**y**, $\hat{x}$) measures the marginal decrease in class *s*′ offspring produced by a class *s* individual owing to expression of the mutant allele in that individual. The benefit term *b*_*s*′*σ*←*s*_(**y**, $\hat{x}$) measures the marginal increase in the number of class *s*′ offspring produced by individuals of class *σ* owing to the mutant allele expression by a groupmate of class *s*. The invasion fitness can then be expressed as (using an *inclusive fitness* or *actor-modulated* approach) [54]:

6.4 $$\lambda (y,\hat{x})=1+\sum_{s^{\prime },s}q_{s}(y,\hat{x})v_{s^{\prime }}(y,\hat{x})\left(-c_{s^{\prime }s}(y,\hat{x})+\sum_{\sigma }b_{s^{\prime }\sigma \leftarrow s}(y,\hat{x})r_{\sigma |s}(y,\hat{x})\right),$$

which is the inclusive fitness [29] of the mutant allele. In equation (6.4), *r*_*σ*|*s*_(**y**, $\hat{x}$) is the genetic relatedness in a group and is the probability that a random groupmate of class *σ* has the mutant allele given an individual of class *s* has the mutant allele, *v*_*s*′_(**y**, $\hat{x}$) is the normalized reproductive value of class *s*′, and *q*_*s*_(**y**, $\hat{x}$) is the probability that a mutant individual in the invasion process is in class *s* and is the sum of *q*_**k**,*s*_(**y**, $\hat{x}$) over **k** (see [54] for technical definitions of these quantities). Equation (6.4) shows how invasion fitness can be partitioned into the direct effects of mutant allele expression, which are cost terms, and the indirect effects, which are the benefit times relatedness terms. Thus, kin selection and the evolution of altruistic traits are easily captured with an ESS analysis of invasion fitness.

Given that previous work using the Price equation has shown that kin selection and group selection partitions of fitness are simply alternative ways of expressing genotype frequency change in a population [20,137,139–141], it should be possible to rewrite invasion fitness so as to group terms that constitute between-group selection, *ω*_*G*,*s*′*s*_(**y**, $\hat{x}$), and those that constitute within-group selection, *ω*_Δ*G*,*s*′*s*_(**y**, $\hat{x}$). In fact, if we apply a *neighbour-modulated* approach from [20,137] to the results from [54], we obtain

6.5 $$\begin{array}{r} \lambda (y,\hat{x})=1+\sum_{s^{\prime },s}q_{s}(y,\hat{x})v_{s^{\prime }}(y,\hat{x})(\omega_{G,s^{\prime }s}(y,\hat{x})+\omega_{\Delta G,s^{\prime }s}(y,\hat{x})), \end{array}$$

where, dropping the dependence on $\hat{x}$ and **y** for ease of presentation:

6.6 $$\begin{array}{rl} & \omega_{G,s^{\prime }s}=(b_{s^{\prime }s\leftarrow s}-c_{s^{\prime }s})\left(\frac{1}{n_{s}}+\frac{n_{s}-1}{n_{s}}r_{s|s}\right)+\sum_{\sigma \neq s}b_{s^{\prime }s\leftarrow \sigma }r_{\sigma |s}\\ \mathrm{and}\; & \omega_{\Delta G,s^{\prime }s}=-(b_{s^{\prime }s\leftarrow s}+c_{s^{\prime }s})\frac{n_{s}-1}{n_{s}}(1-r_{s|s}). \end{array}\}$$

We can see immediately that if mutant allele expression is costly to the individual itself, *c*_*s*′*s*_ > 0, helps others, *b*_*s*′*s*←*σ*_ > 0 for all classes *σ*, and the benefits outweigh the costs within a class, *b*_*s*′*s*←*s*_ − *c*_*s*′*s*_ > 0, then the mutant allele expression is positively selected by between-group selection and negatively selected by within-group selection. Since both the kin selection and group selection partitions of invasion fitness (equations (6.4) and (6.5), respectively) are expressed using the same benefit, cost and relatedness functions, a single ESS analysis using invasion can answer questions derived from both perspectives.

## 7. Variable environments, recombination and evolutionarily stable strategies

One of the important features of Result 4.1 is that it shows how under some genetic and demographic scenarios (i.e. a large, randomly mating population in a constant environment) an ESS phenotype maybe independent of some genetic parameters like the recombination rate between the loci affecting the phenotype. Another example of this kind of result is the ‘reduction principle’ of Feldman, Liberman and colleagues [146–150], which says that for large, randomly mating populations with viability selection in a constant environment, the only uninvadable or unbeatable rates of recombination, mutation and migration are the lowest possible rates (typically zero). Like Result 4.1, the reduction principle is proved by analysing the external stability of an internally stable equilibrium and calculating the invasion fitness of a rare mutant allele. However, alleles at the genetic locus where external stability is tested, which is called the modifier locus, do not directly affect fitness and instead modify rates of recombination, mutation or migration. Given the connection between invasion fitness and ESS analysis, the reduction principle shows that rates of zero recombination, mutation, or migration are strict ESSs. In effect, the reduction principle says natural selection on populations at equilibrium in constant environments acts to reduce genetic and demographic processes that increase genetic variation; this is intuitive as such genetic variation must result in lower fitness genotypes since the population is already at an equilibrium and the environment is constant. Like Result 4.1, the reduction principle holds independent of the recombination rate between the modifier locus and major loci that affect fitness. Since the reduction principle assumes large population size, forces like local competition that might generate positive ESS values of migration are not a factor [144].

**Figure 1.**
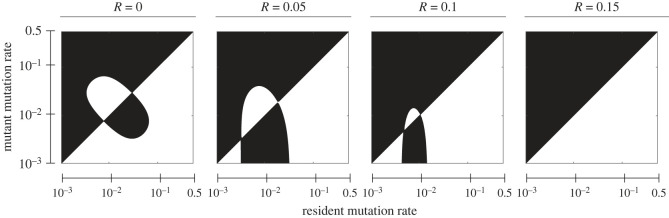
Pairwise invasibility plots for the mutation rate model of [168] when alternation between each of the two environments occurs every *T* = 50 generations. The fitness costs of being in the wrong environments are *s*_1_ = 0.01 and *s*_2_ = 0.015 and the recombination rate between the phenotypic locus and the modifier is given above each plot. White regions are where a mutant modifier allele with a mutation rate given on the vertical axis can invade a population fixed for a modifier allele with a mutation rate given on the horizontal axis, and black regions are where the mutant cannot invade; in other words, the leading eigenvalue of external stability matrix $L_{\mathrm{ex}}$ in eqn. (17) of Liberman *et al*. [168] is greater than one in the white regions and less than one in the black regions.

In environments that are not constant, the reduction principle does not hold as genetic variation can be beneficial depending on the environmental state. Thus, natural selection may favour genetic and demographic mechanisms that generate genetic variation like positive rates of recombination, mutation and migration. In fact, environmental variation, particularly over time, is one of the most well studied and important factors supporting the evolution of genotypic and phenotypic variation through mechanisms that promote such variation like recombination [151–153], mutation [154–156], migration [157–159], phenotypic plasticity [160–163] and bet-hedging [164–167]. Recent work shows that not only are the ES recombination, mutation and migration rates positive in variable environments, but they also depend on genetic parameters like the recombination rate between the major and modifier loci. In the case of mutation rate evolution, Liberman *et al*. [168] used an analysis of the external stability of the mutation rate to show that the ES mutation rate depends on the recombination rate between phenotypic locus and the modifier locus. For example, figure 1 shows pairwise invasibility plots for the mutation rate with different recombination rates and given weakly asymmetric selection in two different environments that alternate every *T* = 50 generations. White regions are where mutant alleles with a mutation rate on the vertical axis can invade a population with a resident mutation rate given by the horizontal axis; black regions are when such a mutant cannot invade. The plots show that a mutation rate of approximately 1/50 is ES when the recombination rate with the modifier locus is zero, similar to older results [154,156] that show the ES mutation rate is approximately 1/*T*. The ES mutation rate decreases as recombination with the modifier locus increases until there is a discontinuous shift to an ES rate of zero switching. Carja *et al.* [169] generalize this approach to the evolution of recombination, mutation and migration in fluctuating environments. They first show that the rate of environmental fluctuation has the same effect on ES recombination and migration rates as it does on ES mutation rates, namely that slower rates of fluctuation lead to slower recombination, mutation and migration rates (see fig. 1 in [169]). In other words, as the rate of environmental change slows, so do the ES rates of genetic and demographic processes that generate genetic variation. Carja *et al.* [169] also show that these ES rates all decrease with increasing rates of recombination between the major and modifier loci (see fig. S5 in [169]). This can be understood as owing to the fact that the strength of indirect selection at the modifier locus decreases as increased recombination erodes linkage disequilibrium between the major and modifier loci.

## 8. Assumptions of long-term evolution and evolutionarily stable strategies

The previous sections show how ESS analysis using invasion fitness can be used to predict the long-term outcomes of phenotypic evolution in scenarios with both genetic and demographic structure. However, the generality of these results ultimately depends on the assumptions made in developing Result 4.1 in §4, which relates the ESS condition to equilibria of the long-term evolution process. These assumptions are^7^:

(i) phenotypes are probabilities of selecting among *two* behaviours or strategies;
(ii) fitness (i.e. pay-off) is a linear function of phenotype;
(iii) individuals interact in pairs that form randomly;
(iv) populations are large enough to ignore stochastic forces including genetic drift;
(v) mutations are infrequent enough so that a new mutation reaches a short-term equilibrium before additional mutations occur; and
(vi) phenotypes are determined by two (or more) haploid or diploid loci.

Some of these of these assumptions are probably less restrictive than others. For example, assumption (vi) may not be very limiting as many organisms are haploid or diploid and ESS models of other genetic systems like haplodiploidy can be studied by adding demographic structure using matrix population models [144,170,171] like the one described in §6 on ESS and inclusive fitness. The large population size assumption (iv) also can be relaxed by using stochastic population models that allow for genetic drift and use fixation probability as a fitness measure instead of invasion fitness [107,172–175]. Assumption (v) is more difficult to relax since doing so allows multiple mutants to occur at a time. Analysis of such multi-mutant invasions is beyond the scope of the mathematical tools typically used in evolutionary analysis. One potential route forward for such analyses might be to follow Luo and Cooney who used non-local integro-differential partial differential equations to study the evolution of cooperation via multilevel selection [47,176–179].

Assumption (i) that there are two behaviours or strategies is particularly important. In practice, games like rock paper scissors [89,91,102,180] involving three or more strategies may have more complex evolutionary dynamics resulting in equilibria that may or may not be ESSs. Support for this possibility comes from examples of three strategy interactions like rock paper scissors where the ESS strategy is actually an internally *unstable* equilibrium [180,181]. Weissing sees this issue as a potentially serious limitation for using ESS to describe long-term phenotypic evolution [101]. Interestingly, much of the mathematical machinery developed for analysing external stability can be generalized to three or more strategies (e.g. [95,101,102,106]), and ESS is an appropriate determinant for stability for some three strategy population genetic models (e.g. single-locus three-allele models in continuous time [98]). Thus, future theoretical work might yield conditions under which the two strategy assumption can be relaxed.

The linear fitness function assumption (ii) is also an important restriction. It arises naturally from assuming that the fitness of an individual using a mixed strategy phenotype is the expected fitness that individual would obtain using pure strategies with probabilities given by the mixed strategy. Linear fitness functions are convenient in part because they simplify interactions involving more than two individuals: fitness in such interactions can be expressed as a linear combination of the fitnesses of pairwise interactions. This means the pairwise interaction assumption (iii) is unnecessary for linear fitness functions. However, phenotypes often involve continuous quantities like body size or metabolic rate and nonlinear fitness functions are the norm in these cases. Studying nonlinear fitness functions, Eshel, Taylor and others [115,116,182–186] showed that evolutionary stability involves two qualitatively different kinds of stability. The first kind is the ability of a population with mean phenotype x* to resist invasion by any mutant genotypes with phenotype not equal to x*, which is the condition captured by the strict ESS condition (2.1*a*). This is the classic notion of evolutionary stability evoked by verbal descriptions of the ESS concept. The second kind of stability is called *convergence stability* [99,116,185].

## 9. Convergence of long-term evolution and evolutionarily stable strategies

Convergence stability refers to populations with mean phenotype *x* near an ESS x* that are invaded by mutants that bring the population closer to x* and that resist invasion by mutants that move the population further from x* [183,184]. Thus, populations near a convergence stable phenotype x* will evolve towards x*. If an ESS x* is to really represent the outcome of a long-term evolution process, then it should be convergence stable as well as an ESS. The evolutionary and convergence stability conditions for continuous phenotypes and nonlinear fitness functions are expressed in terms of partial derivatives of the fitness function with respect to the mutant and resident phenotypes [116,183,184]. However, we can still get a sense of a formal statement of the convergence stability condition without the partial derivative expressions by examining the first inequality in the weak ESS condition (2.1*b*), *w*(x*, *y*) > *w*(*y*, *y*), which says that x* should invade a population with an alternative phenotype *y*. In a sense then, the original Maynard Smith & Price definition of an ESS [2,5] contained the notion of convergence stability but did not distinguish it from evolutionary stability more generally. Only by examining continuous phenotypes and nonlinear fitness functions did convergence stability emerge clearly as an essential and distinct condition.

Given there are two kinds of stability to assess, evolutionary stability *sensu* condition (2.1*a*) and convergence stability, a phenotype x* that is not long-term stable might fail either one of them. If x* is an ESS but not convergence stable, then a population with mean phenotype x* will resist invasion by mutants with alternative phenotypes but populations with mean phenotypes near x* will eventually evolve away from x*. Such ESSs are unattainable under long-term evolution and are sometimes called ‘Garden of Eden’ phenotypes because no predecessor population could evolve towards them [187]. Even more interesting are the cases where the phenotype x* is convergence stable but not an ESS. This possibility was not explored much before Metz, Geritz, Kisdi and colleagues laid out in detail how such cases should lead to the evolution of phenotypic polymorphisms as the population mean approaches x* [115,116]. This work spawned the field of *adaptive dynamics* [117] where the emergence of phnenotypic polymorphism at convergence stable points x* that are not ESSs is called ‘evolutionary branching’ since the polymorphism evolves as a dimorphism of phenotypes on either side of x* in phenotype space [115,116]. Studies of evolutionary branching have been important for theories of adaptive speciation [188,189], evolutionary rescue [190] and other areas of evolutionary ecology [117]. While evolutionary branching reveals the limits of long-term evolution since long-term evolution can only predict stable monomorphic phenotypes, the long-term process is still an important tool as it identifies where these branching points occur so that further analysis can study the evolution of the resulting phenotypic diversity.

## 10. Conclusion and the future of evolutionarily stable strategy analysis

When Maynard Smith & Price [2,5] introduced ESS analysis, the revolutions in molecular genetics and genomics were still two and three decades off, respectively, and evolutionary biology was being roiled by questions and debates regarding the role of natural selection in the evolution of phenotypic novelty broadly [9] and in the evolution of animal and human behaviour specifically [191,192]. These debates drew sharp lines between proponents of evolutionary theory derived from explicit genetic and demographic assumptions and theories of phenotypic change based on natural selection alone, namely fitness optimization and ESS analysis. Even among those who agreed that ESS analysis was an important approach, questions remained about what measure of fitness to optimize, individual or inclusive fitness and how to incorporate multilevel selection. In the decades following Maynard Smith & Price [2] however, mathematical analysis bridged some of the division by showing how ESSs can be viewed as the evolutionary attractors of a long-term evolutionary process that builds on short-term evolutionary change owing to natural selection, mutation, recombination and other evolutionary forces. Mathematical analysis of the long-term process also revealed that invasion fitness as a measure of external stability is the appropriate way to measure evolutionary success and that it can incorporate kin and group selection effects in group structured populations.

One of the initial goals of the long-term evolution approach was to determine the degree to which ESS analyses can be independent of the underlying genetics and demography of populations. Put another way, how justifiable is the phenotypic gambit? Result 4.1 shows that for large and randomly mating populations, ESSs can be fairly independent of genetic parameters like mutation and recombination rates and even the number of loci. At the time, this independence was important for supporting the use of ESS analysis across a broad range of species with different genetics and demography. However, this independence may no longer be as useful given the growing abundance of genomic, transcriptomic and long-term demographic data for natural populations. Instead, we might rather want to make use of these data to refine our evolutionary models, and the ESS may be a less useful approach if it cannot take advantage of these data. However, long-term evolution and consequently ESSs are in fact sensitive to genetics and demography for many traits and populations more complex than Result 4.1 assumes. The threshold for such complexity can be quite low as in the case of sex ratio evolution where details of the genetic system can be crucial [121,125,193,194], in the case of kin and group structured populations where demography determines the fate of genes with social effects, and in the case of recombination, mutation and migration rate evolution in variable environments where the ES rates depend on the recombination rate between the major and modifier loci.

All of the ESS analysis so far has concerned a single phenotypic trait. However, the principle of invasion fitness can also be applied to multiple coevolving traits [195–197]. When multiple traits coevolve, issues of pleiotropy and genetic constraint immediately arise as mutations may not cause independent effects along the different phenotypic dimensions. Thus, even in the case of two coevolving traits, genetic constraints owing to the underlying genetic architecture can have strong effects on long-term evolution and the resulting ESSs. The study of coevolving traits is nothing new to quantitative genetics [198–200] where the importance of genetic constraints has long been emphasized. In fact, recent work by Mullon & Lehmann [197] ties together the quantitative genetic and invasion fitness approaches for coevolving traits. By building from an invasion fitness perspective that is comfortable with fitness as a complex function of species ecology and behaviour, this synthetic approach shows how pleiotropy, demography and behaviour can interact to shape the coevolution of two synergistic social traits [197].

Many conceptual divides in evolutionary theory have been bridged in recent decades including divides surrounding ESS analysis and its role in genotypic and phenotypic evolution and in kin and group selection. Given these advances, it is hard to be pessimistic about the prospect that evolutionary theory can tackle big open questions about the emergence and persistence of biodiversity. Even questions with global consequences like climate change, biodiversity loss and global conflict may yet be advanced by a more unified evolutionary theory that can address the simultaneous action of genetic, phenotypic, social and cultural change.

## Data Availability

MATLAB code for generating figure 1 is available on Zenodo: https://doi.org/10.5281/zenodo.7644630.

## Endnotes

1 The marginal fitness of a genotype or allele is the mean fitness of that genotype or allele among all contexts in which it occurs in the population. For example, the marginal fitness of one allele at a single diploid locus in the mean fitness of that allele in combination with all other alleles according to their frequencies.

2 When invasion occurs at more complex attractors like cycles, the *invasion exponent* is used, which is log|*λ*| [122].

3 This is also referred to as an *evolutionary invasion analysis*.

4 The relationship between invasion fitness and non-strict ESSs is more complex and depends on specific details of the genetics of the population. For example, see the section entitled ‘Long-term Stability of an ESS’ in [106].

5 See also the discussion of ‘evolutionary genetic stability’ in [99, pp. 505–506].

6 Marginal effects in this context are additive effects of the phenotype on fitness and are often measured with partial regression coefficients.

7 For additional discussion of these assumptions, see [99–101].

## References

[1] Lewontin RC. 1961 Evolution and the theory of games. J. Theor. Biol. **1**, 382-403. (10.1016/0022-5193(61)90038-8) 13761767

[2] Maynard Smith J, Price GR. 1973 The logic of animal conflict. Nature **246**, 15-18.

[3] Myerson RB. 1991 Game theory: analysis of conflict. Cambridge, MA: Harvard University Press.

[4] Nash JF. 1950 Equilibrium points in n-person games. Proc. Natl Acad. Sci. USA **36**, 48-49. (10.1073/pnas.36.1.48) 16588946 PMC1063129

[5] Maynard Smith J. 1974 The theory of games and the evolution of animal conflicts. J. Theor. Biol. **47**, 209-221. (10.1016/0022-5193(74)90110-6) 4459582

[6] Maynard Smith J, Parker GA. 1976 The logic of asymmetric contests. Anim. Behav. **24**, 159-175.

[7] Maynard Smith J. 1982 Evolution and the theory of games. Cambridge, UK: Cambridge University Press.

[8] Maynard Smith J. 1978 Optimization theory in evolution. Annu. Rev. Ecol. Syst. **9**, 31-56.

[9] Gould SJ, Lewontin RC. 1979 The spandrels of San Marco and the Panglossian paradigm: a critique of the adaptationist programme. Proc. R. Soc. B **205**, 581-598. (10.1098/rspb.1979.0086) 42062

[10] Lewontin RC. 1979 Sociobiology as an adaptationist program. Behav. Sci. **24**, 5-14. (10.1002/bs.3830240103) 435219

[11] Orzack SH, Sober E. 1994 Optimality models and the test of adaptationism. Am. Nat. **143**, 361-380. (10.1086/285608)

[12] Gardner A. 2017 The purpose of adaptation. Interface Focus **7**, 20170005. (10.1098/rsfs.2017.0005) 28839927 PMC5566815

[13] Kern AD, Hahn MW. 2018 The neutral theory in light of natural selection. Mol. Biol. Evol. **35**, 1366-1371. (10.1093/molbev/msy092) 29722831 PMC5967545

[14] Jensen JD, Payseur BA, Stephan W, Aquadro CF, Lynch M, Charlesworth D, Charlesworth B. 2019 The importance of the neutral theory in 1968 and 50 years on: a response to Kern and Hahn 2018. Evolution **73**, 111-114. (10.1111/evo.13650) 30460993 PMC6496948

[15] Hamilton WD. 1963 The evolution of altruistic behavior. Am. Nat. **97**, 354-356. (10.1086/497114)

[16] Maynard Smith J. 1964 Group selection and kin selection. Nature **201**, 1145-1147.

[17] Price GR. 1972 Extension of covariance selection mathematics. Ann. Hum. Genet. **35**, 485-490. (10.1111/j.1469-1809.1957.tb01874.x) 5073694

[18] Wilson DS, Wilson EO. 2007 Rethinking the theoretical foundation of sociobiology. Q. Rev. Biol. **82**, 327-348. (10.1086/522809) 18217526

[19] Leigh Jr EG. 2010 The group selection controversy. J. Evol. Biol. **23**, 6-19. (10.1111/j.1420-9101.2009.01876.x) 20002254

[20] Akçay E, Van Cleve J. 2012 Behavioral responses in structured populations pave the way to group optimality. Am. Nat. **179**, 257-269. 22218314 10.1086/663691

[21] West SA, Griffin AS, Gardner A. 2007 Social semantics: altruism, cooperation, mutualism, strong reciprocity and group selection. J. Evol. Biol. **20**, 415-432. (10.1111/j.1420-9101.2006.01258.x) 17305808

[22] Gardner A, Grafen A. 2009 Capturing the superorganism: a formal theory of group adaptation. J. Evol. Biol. **22**, 659-71. (10.1111/j.1420-9101.2008.01681.x) 19210588

[23] Nowak MA, Tarnita CE, Wilson EO. 2010 The evolution of eusociality. Nature **466**, 1057-1062. (10.1038/nature09205) 20740005 PMC3279739

[24] Abbot P et al. 2011 Inclusive fitness theory and eusociality. Nature **471**, E1-E4; author reply E9–10. (10.1038/nature09831) 21430721 PMC3836173

[25] Allen B, Nowak MA, Wilson EO. 2013 Limitations of inclusive fitness. Proc. Natl Acad. Sci. USA **110**, 20135-20139. (10.1073/pnas.1317588110) 24277847 PMC3864293

[26] Birch J. 2014 Hamilton’s rule and its discontents. Br. J. Phil. Sci. **65**, 381-411. (10.1093/bjps/axt016)

[27] Birch J. 2017 The inclusive fitness controversy: finding a way forward. R. Soc. Open Sci. **4**, 170335. (10.1098/rsos.170335) 28791162 PMC5541557

[28] Nowak MA, McAvoy A, Allen B, Wilson EO. 2017 The general form of Hamilton’s rule makes no predictions and cannot be tested empirically. Proc. Natl Acad. Sci. USA **114**, 5665-5670. (10.1073/pnas.1701805114) 28512224 PMC5465884

[29] Hamilton WD. 1964 The genetical evolution of social behaviour. I. J. Theor. Biol. **7**, 1-16. (10.1016/0022-5193(64)90038-4) 5875341

[30] Lewontin RC. 1970 The units of selection. Annu. Rev. Ecol. Syst. **1**, 1-18. (10.1146/annurev.es.01.110170.000245)

[31] Dawkins R. 1982 The extended phenotype. Oxford, UK: Oxford University Press.

[32] Wilson DS, Sober E. 1989 Reviving the superorganism. J. Theor. Biol. **136**, 337-356. (10.1016/S0022-5193(89)80169-9) 2811397

[33] Maynard Smith J, Szathmáry E. 1995 The major transitions in evolution. Oxford, UK: Oxford University Press.

[34] Wilson DS. 1997 Altruism and organism: disentangling the themes of multilevel selection theory. Am. Nat. **150**(Suppl 1), S122-S134. (10.1086/286053) 18811309

[35] Michod RE. 1999 Darwinian dynamics. Princeton, NJ: Princeton University Press.

[36] Michod RE. 2006 The group covariance effect and fitness trade-offs during evolutionary transitions in individuality. Proc. Natl Acad. Sci. USA **103**, 9113-9117. (10.1073/pnas.0601080103) 16751277 PMC1482575

[37] Szathmáry E. 2015 Toward major evolutionary transitions theory 2.0. Proc. Natl Acad. Sci. USA **112**, 10 104-10 111. (10.1073/pnas.1421398112) 25838283 PMC4547294

[38] Hull DL. 1980 Individuality and selection. Annu. Rev. Ecol. Syst. **11**, 311-332. (10.1146/annurev.es.11.110180.001523)

[39] Brandon R. 1982 The levels of selection. In *PSA Proc. Bienn. Meet. Philos. Sci. Assoc.*, vol. 1 (eds PD Asquith, T Nickles), pp. 315–323. Cambridge, UK: Cambridge University Press.

[40] Damuth J, Heisler IL. 1988 Alternative formulations of multilevel selection. Biol. Phil. **3**, 407-430. (10.1007/BF00647962)

[41] Lloyd EA. 1992 Unit of selection. In *Keywords in evolutionary biology* (eds EF Keller, EA Lloyd), pp. 334–340. Cambridge, MA: Harvard University Press.

[42] Lloyd EA. 1994 The structure and confirmation of evolutionary theory. Princeton, NJ: Princeton University Press.

[43] Sober E, Wilson DS. 1994 A critical review of philosophical work on the units of selection problem. Phil. Sci. **61**, 534-555. (10.1086/289821)

[44] Okasha S. 2006 Evolution and the levels of selection. Oxford, UK: Oxford University Press.

[45] Okasha S. 2016 The relation between kin and multilevel selection: an approach using causal graphs. Br. J. Phil. Sci. **67**, 435-470. (10.1093/bjps/axu047)

[46] Black AJ, Bourrat P, Rainey PB. 2020 Ecological scaffolding and the evolution of individuality. Nat. Ecol. Evol. **4**, 426-436. (10.1038/s41559-019-1086-9) 32042121

[47] Cooney DB, Mori Y. 2022 Long-time behavior of a PDE replicator equation for multilevel selection in group-structured populations. J. Math. Biol. **85**, 12. (10.1007/s00285-022-01776-6) 35864421

[48] Veit W. 2022 Scaffolding natural selection. Biol. Theory **17**, 163-180. (10.1007/s13752-021-00387-6)

[49] Metz JAJ, Nisbet RM, Geritz SAH. 1992 How should we define ‘fitness’ for general ecological scenarios? Trends Ecol. Evol. **7**, 198-202. (10.1016/0169-5347(92)90073-K) 21236007

[50] Heino M, Metz JAJ, Kaitala V. 1998 The enigma of frequency-dependent selection. Trends Ecol. Evol. **13**, 367-370. (10.1016/S0169-5347(98)01380-9) 21238345

[51] Lehmann L, Alger I, Weibull J. 2015 Does evolution lead to maximizing behavior? Evolution **69**, 1858-1873. (10.1111/evo.12701) 26082379

[52] Akçay E, Van Cleve J. 2016 There is no fitness but fitness, and the lineage is its bearer. Phil. Trans. R. Soc. B **371**, 20150085. (10.1098/rstb.2015.0085) 26729925 PMC4760187

[53] Lehmann L, Mullon C, Akçay E, Van Cleve J. 2016 Invasion fitness, inclusive fitness, and reproductive numbers in heterogeneous populations. Evolution **70**, 1689-1702. (10.1111/evo.12980) 27282317

[54] Lehmann L, Rousset F. 2020 When do individuals maximize their inclusive fitness? Am. Nat. **195**, 717-732. (10.1086/707561) 32216664

[55] Fisher RA. 1930 The genetical theory of natural selection, 2nd edn. Oxford, UK: The Clarendon Press.

[56] Wright S. 1955 Classification of the factors of evolution. Cold Spring Harb. Symp. Quant. Biol. **20**, 16-24. (10.1101/SQB.1955.020.01.004) 13433551

[57] Kojima KI, Kelleher TM. 1961 Changes of mean fitness in random mating populations when epistasis and linkage are present. Genetics **46**, 527-540. (10.1093/genetics/46.5.527) 13757645 PMC1210217

[58] Moran PAP. 1964 On the nonexistence of adaptive topographies. Ann. Hum. Genet. **27**, 383-393. (10.1111/j.1469-1809.1963.tb01535.x) 14175202

[59] Karlin S. 1975 General two-locus selection models: some objectives, results and interpretations. Theor. Popul. Biol. **7**, 364-398. (10.1016/0040-5809(75)90025-8) 1179266

[60] Akin E. 1979 The geometry of population genetics. *No. 31.* *Lecture notes in biomathematics*. Berlin, Heidelberg, Germany: Springer.

[61] Zuckerkandl E, Pauling L. 1965 Evolutionary divergence and convergence in proteins. In *Evolving genes and proteins* (eds V Bryson, HJ Vogel), pp. 97–166. New York, NY: Academic Press. 10.1016/0022-5193(65)90083-4 5876245

[62] King JL, Jukes TH. 1969 Non-Darwinian evolution. Science **164**, 788-798. (10.1126/science.164.3881.788) 5767777

[63] Harris H. 1966 Enzyme polymorphisms in man. Proc. R. Soc. Lond. B **164**, 298-310. (10.1098/rspb.1966.0032) 4379519

[64] Lewontin RC, Hubby JL. 1966 A molecular approach to the study of genic heterozygosity in natural populations. II. Amount of variation and degree of heterozygosity in natural populations of *Drosophila pseudoobscura*. Genetics **54**, 595-609. (10.1093/genetics/54.2.595) 5968643 PMC1211186

[65] Kimura M. 1968 Evolutionary rate at the molecular level. Nature **217**, 624-626. (10.1038/217624a0) 5637732

[66] Kimura M. 1983 The neutral theory of molecular evolution. Cambridge, UK: Cambridge University Press.

[67] Ohta T. 1974 Mutational pressure as the main cause of molecular evolution and polymorphism. Nature **252**, 351-354. (10.1038/252351a0) 4610412

[68] Ohta T. 1992 The nearly neutral theory of molecular evolution. Annu. Rev. Ecol. Syst. **23**, 263-286. (10.1146/annurev.es.23.110192.001403)

[69] Ohta T, Gillespie JH. 1996 Development of neutral and nearly neutral theories. Theor. Popul. Biol. **49**, 128-142. (10.1006/tpbi.1996.0007) 8813019

[70] Gillespie JH, Langley CH. 1974 A general model to account for enzyme variation in natural populations. Genetics **76**, 837-848. (10.1093/genetics/76.4.837) 4838763 PMC1213108

[71] Gillespie JH. 1978 A general model to account for enzyme variation in natural populations. V. The SAS-CFF model. Theor. Popul. Biol. **14**, 1-45. (10.1016/0040-5809(78)90002-3) 741392

[72] Grafen A. 1984 Natural selection, kin selection and group selection. In *Behavioural ecology: an evolutionary approach* (eds JR Krebs, NB Davies), 2nd edn, pp. 62–84. Oxford, UK: Blackwell Scientific Publications.

[73] Houston AI, McNamara JM. 1999 Models of adaptive behaviour. Cambridge, UK: Cambridge University Press.

[74] Kapheim KM et al. 2015 Genomic signatures of evolutionary transitions from solitary to group living. Science **348**, 1139-1143. (10.1126/science.aaa4788) 25977371 PMC5471836

[75] Mikheyev AS, Linksvayer TA. 2015 Genes associated with ant social behavior show distinct transcriptional and evolutionary patterns. eLife **4**, e04775. (10.7554/eLife.04775) 25621766 PMC4383337

[76] Warner MR, Mikheyev AS, Linksvayer TA. 2017 Genomic signature of kin selection in an ant with obligately sterile workers. Mol. Biol. Evol. **34**, 1780-1787. (10.1093/molbev/msx123) 28419349 PMC5455959

[77] Kocher SD, Mallarino R, Rubin BER, Yu DW, Hoekstra HE, Pierce NE. 2018 The genetic basis of a social polymorphism in halictid bees. Nat. Commun. **9**, 4338. (10.1038/s41467-018-06824-8) 30337532 PMC6194137

[78] Warner MR, Mikheyev AS, Linksvayer TA. 2019 Transcriptomic basis and evolution of the ant nurse-larval social interactome. PLoS Genet. **15**, e1008156. (10.1371/journal.pgen.1008156) 31107868 PMC6544314

[79] Springer SA, Crespi BJ, Swanson WJ. 2011 Beyond the phenotypic gambit: molecular behavioural ecology and the evolution of genetic architecture. Mol. Ecol. **20**, 2240-2257. (10.1111/j.1365-294X.2011.05116.x) 21507096

[80] Rittschof CC, Robinson GE. 2014 Genomics: moving behavioural ecology beyond the phenotypic gambit. Anim. Behav. **92**, 263-270. (10.1016/j.anbehav.2014.02.028) 24954950 PMC4061759

[81] Akçay E, Linksvayer TA, Van Cleve J. 2015 Bridging social evolution theory and emerging empirical approaches to social behavior. Curr. Opin. Behav. Sci. **6**, 59-64. (10.1016/j.cobeha.2015.09.002)

[82] Cunningham CB. 2020 Functional genomics of parental care of insects. Horm. Behav. **122**, 104756. (10.1016/j.yhbeh.2020.104756) 32353447

[83] Osborne MJ, Rubinstein A. 1994 A course in game theory. Cambridge, MA: MIT Press.

[84] Bishop DT, Cannings C. 1976 Models of animal conflict. Adv. Appl. Probab. **8**, 616-621. (10.2307/1425917)

[85] Rapoport A, Chammah AM. 1965 Prisoner’s Dilemma: a study in conflict and cooperation. Ann Arbor, MI: University of Michigan Press.

[86] Sugden R. 1986 The economics of rights, co-operation, and welfare. Oxford, UK: Blackwell.

[87] Crow JF, Kimura M. 1970 An introduction to population genetics theory. New York, NY: Harper & Row.

[88] Ewens WJ. 2004 Mathematical population genetics. *No. 27.* *Interdisciplinary applied mathematics*. New York, NY: Springer.

[89] Taylor PD, Jonker LB. 1978 Evolutionary stable strategies and game dynamics. Math. Biosci. **40**, 145-156. (10.1016/0025-5564(78)90077-9)

[90] Hofbauer J, Schuster P, Sigmund K. 1979 A note on evolutionary stable strategies and game dynamics. J. Theor. Biol. **81**, 609-612. (10.1016/0022-5193(79)90058-4) 537389

[91] Zeeman EC. 1980 Population dynamics from game theory. In *Global theory of dnamical systems. Lecture notes in mathematics* (eds Z Nitecki, C Robinson), pp. 471–497. Berlin, Germany: Springer.

[92] Eshel I. 1982 Evolutionarily stable strategies and viability selection in mendelian populations. Theor. Popul. Biol. **22**, 204-217. (10.1016/0040-5809(82)90042-9) 16271737

[93] Hofbauer J, Schuster P, Sigmund K. 1982 Game dynamics in Mendelian populations. Biol. Cybern. **43**, 51-57. (10.1007/BF00337287)

[94] Cressman R, Hines WGS. 1984 Evolutionarily stable strategies of diploid populations with semi-dominant inheritance patterns. J. Appl. Probab. **21**, 1-9. (10.2307/3213659)

[95] Eshel I, Feldman MW. 1984 Initial increase of new mutants and some continuity properties of ESS in two-locus systems. Am. Nat. **124**, 631-640. (10.1086/284303)

[96] Lessard S. 1984 Evolutionary dynamics in frequency-dependent two-phenotype models. Theor. Popul. Biol. **25**, 210-234. (10.1016/0040-5809(84)90019-4) 6729752

[97] Cressman R. 1988 Frequency-dependent viability selection (a single-locus, multi-phenotype model). J. Theor. Biol. **130**, 147-165. (10.1016/S0022-5193(88)80090-0) 3419178

[98] Cressman R, Hofbauer J, Hines WGS. 1996 Evolutionary stability in strategic models of single-locus frequency-dependent viability selection. J. Math. Biol. **34**, 707-733.

[99] Eshel I. 1996 On the changing concept of evolutionary population stability as a reflection of a changing point of view in the quantitative theory of evolution. J. Math. Biol. **34**, 485-510. (10.1007/BF02409747) 8691082

[100] Hammerstein P. 1996 Darwinian adaptation, population genetics and the streetcar theory of evolution. J. Math. Biol. **34**, 511-532. (10.1007/BF02409748) 8691083

[101] Weissing FJ. 1996 Genetic versus phenotypic models of selection: can genetics be neglected in a long-term perspective? J. Math. Biol. **34**, 533-555. (10.1007/BF02409749) 8691084

[102] Hofbauer J, Sigmund K. 1998 Evolutionary games and population dynamics. Cambridge, UK: Cambridge University Press.

[103] Sigmund K. 1987 A maximum principle for frequency dependent selection. Math. Biosci. **84**, 189-195. (10.1016/0025-5564(87)90091-5)

[104] Liberman U. 1988 External stability and ESS: criteria for initial increase of new mutant allele. J. Math. Biol. **26**, 477-485. (10.1007/BF00276375) 3199045

[105] Eshel I. 1991 Game theory and population dynamics in complex genetical systems: the role of sex in short term and in long term evolution. In *Game equilibrium models I: evolution and game dynamics* (ed. R Selten), pp. 6–28. Berlin, Germany: Springer.

[106] Eshel I, Feldman MW, Bergman A. 1998 Long-term evolution, short-term evolution, and population genetic theory. J. Theor. Biol. **191**, 391-396. (10.1006/jtbi.1997.0597)

[107] Van Cleve J. 2015 Social evolution and genetic interactions in the short and long term. Theor. Popul. Biol. **103**, 2-26. (10.1016/j.tpb.2015.05.002) 26003630

[108] Van Cleve J. 2020 Building a synthetic basis for kin selection and evolutionary game theory using population genetics. Theor. Popul. Biol. **133**, 65-70. (10.1016/j.tpb.2020.03.001) 32165158

[109] Gillespie JH. 1991 The causes of molecular evolution. New York, NY: Oxford University Press.

[110] Champagnat N. 2006 A microscopic interpretation for adaptive dynamics trait substitution sequence models. Stoch. Process. Their Appl. **116**, 1127-1160. (10.1016/j.spa.2006.01.004)

[111] Champagnat N, Ferrière R, Méléard S. 2006 Unifying evolutionary dynamics: from individual stochastic processes to macroscopic models. Theor. Popul. Biol. **69**, 297-321. (10.1016/j.tpb.2005.10.004) 16460772

[112] Desai MM, Fisher DS. 2007 Beneficial mutation selection balance and the effect of linkage on positive selection. Genetics **176**, 1759-98. (10.1534/genetics.106.067678) 17483432 PMC1931526

[113] Dieckmann U, Law R. 1996 The dynamical theory of coevolution: a derivation from stochastic ecological processes. J. Math. Biol. **34**, 579-612. (10.1007/BF02409751) 8691086

[114] Champagnat N, Ferrière R, Ben Arous G. 2001 The canonical equation of adaptive dynamics: a mathematical view. Selection **2**, 73-83. (10.1556/Select.2.2001.1-2.6)

[115] Metz JAJ, Geritz SAH, Meszéna G, Jacobs FJA, van Heerwaarden JS. 1996 Adaptive dynamics, a geometrical study of the consequences of nearly faithful reproduction. In *Stochastic and spatial structures of dynamical systems, vol. 45 of Konink. Nederl. Akad. Wetensch. Verh. Afd. Natuurk. Eerste Reeks* (eds SJ van Strien, SM Verduyn Lunel), pp. 183–231. Amsterdam, The Netherlands: North-Holland.

[116] Geritz SAH, Metz JAJ. 1998 Evolutionarily singular strategies and the adaptive growth and branching of the evolutionary tree. Evol. Ecol. **12**, 35-57. (10.1023/A:1006554906681)

[117] Dercole F, Rinaldi S. 2008 Analysis of evolutionary processes: the adaptive dynamics approach and its applications. Princeton, NJ: Princeton University Press.

[118] Hammerstein P, Selten R. 1994 Game theory and evolutionary biology. In *Handbook of game theory with economic applications, vol. 2 of Handbooks in economics* (eds R Aumann, S Hart), pp. 929–993. Amsterdam, The Netherlands: Elsevier.

[119] Edelstein-Keshet L. 2005 Mathematical models in biology, *vol. 46*. *Classics in applied mathematics*. Philadelphia, PA: Society for Industrial and Applied Mathematics.

[120] Caswell H. 2006 Matrix population models: construction, analysis, and interpretation, 2nd edn. Sunderland, MA: Sinauer.

[121] Hamilton WD. 1967 Extraordinary sex ratios. Science **156**, 477-488. (10.1126/science.156.3774.477) 6021675

[122] Rand DA, Wilson HB, McGlade JM. 1994 Dynamics and evolution: evolutionarily stable attractors, invasion exponents and phenotype dynamics. Phil. Trans. R. Soc. Lond. B **343**, 261-283. (10.1098/rstb.1994.0025) 8066105

[123] Fisher RA. 1958 The genetical theory of natural selection. New York, NY: Dover.

[124] Eshel I, Feldman MW. 1982 On evolutionary genetic stability of the sex ratio. Theor. Popul. Biol. **21**, 430-439. (10.1016/0040-5809(82)90028-4)

[125] Eshel I, Feldman MW. 1982 On the evolution of sex determination and the sex ratio in haplodiploid populations. Theor. Popul. Biol. **21**, 440-450. (10.1016/0040-5809(82)90029-6)

[126] Wright S. 1943 Isolation by distance. Genetics **28**, 114-138. (10.1093/genetics/28.2.114) 17247074 PMC1209196

[127] Wright S. 1951 The genetical structure of populations. Ann. Eugen. **15**, 323-354. (10.1111/j.1469-1809.1949.tb02451.x) 24540312

[128] Hamilton WD. 1970 Selfish and spiteful behaviour in an evolutionary model. Nature **228**, 1218-1220. (10.1038/2281218a0) 4395095

[129] Rousset F. 2004 Genetic structure and selection in subdivided populations. *No. 40.* *Monographs in population biology*. Princeton, NJ: Princeton University Press.

[130] Lehmann L, Rousset F. 2014 The genetical theory of social behaviour. Phil. Trans. R. Soc. B **369**, 20130357. (10.1098/rstb.2013.0357) 24686929 PMC3982659

[131] Hamilton WD. 1975 Innate social aptitudes of man: an approach from evolutionary genetics. In *Biosocial anthropology* (ed. A Fox), pp. 133–155. London, UK: Malaby Press.

[132] Wilson DS, Pollock GB, Dugatkin LA. 1992 Can altruism evolve in purely viscous populations? Evol. Ecol. **6**, 331-341. (10.1007/BF02270969)

[133] Traulsen A, Nowak MA. 2006 Evolution of cooperation by multilevel selection. Proc. Natl Acad. Sci. USA **103**, 10 952-10 955. (10.1073/pnas.0602530103) 16829575 PMC1544155

[134] Gardner A, West SA. 2006 Demography, altruism, and the benefits of budding. J. Evol. Biol. **19**, 1707-1716. (10.1111/j.1420-9101.2006.01104.x) 16911000

[135] Lehmann L, Rousset F. 2010 How life history and demography promote or inhibit the evolution of helping behaviours. Phil. Trans. R. Soc. B **365**, 2599-2617. (10.1098/rstb.2010.0138) 20679105 PMC2936172

[136] Price GR. 1970 Selection and covariance. Nature **227**, 520-521. (10.1038/227520a0) 5428476

[137] Queller DC. 1992 Quantitative genetics, inclusive fitness, and group selection. Am. Nat. **139**, 540-558. (10.1086/285343)

[138] Lehmann L, Keller L, West S, Roze D. 2007 Group selection and kin selection: two concepts but one process. Proc. Natl Acad. Sci. USA **104**, 6736-6739. (10.1073/pnas.0700662104) 17416674 PMC1871855

[139] Bijma P, Wade MJ. 2008 The joint effects of kin, multilevel selection and indirect genetic effects on response to genetic selection. J. Evol. Biol. **21**, 1175-1188. (10.1111/j.1420-9101.2008.01550.x) 18547354

[140] Gardner A, West SA, Barton NH. 2007 The relation between multilocus population genetics and social evolution theory. Am. Nat. **169**, 207-226. (10.1086/510602) 17211805

[141] Marshall JAR. 2011 Group selection and kin selection: formally equivalent approaches. Trends Ecol. Evol. **26**, 325-32. (10.1016/j.tree.2011.04.008) 21620513

[142] Wright S. 1931 Evolution in mendelian populations. Genetics **16**, 97-159. (10.1093/genetics/16.2.97) 17246615 PMC1201091

[143] Kimmel M, Axelrod DE. 2015 Branching processes in biology, 2nd edn. *No. 19.* *Interdisciplinary applied mathematics*. New York, NY: Springer.

[144] Frank SA. 1998 Foundations of social evolution. Princeton, NJ: Princeton University Press.

[145] Lehmann L, Keller L. 2006 The evolution of cooperation and altruism–a general framework and a classification of models. J. Evol. Biol. **19**, 1365-1376. (10.1111/j.1420-9101.2006.01119.x) 16910958

[146] Feldman MW, Liberman U. 1986 An evolutionary reduction principle for genetic modifiers. Proc. Natl Acad. Sci. USA **83**, 4824-4827. (10.1073/pnas.83.13.4824) 3460074 PMC323834

[147] Liberman U, Feldman MW. 1986 A general reduction principle for genetic modifiers of recombination. Theor. Popul. Biol. **30**, 341-371. (10.1016/0040-5809(86)90040-7) 3810506

[148] Liberman U, Feldman MW. 1986 Modifiers of mutation rate: a general reduction principle. Theor. Popul. Biol. **30**, 125-142. (10.1016/0040-5809(86)90028-6) 3750215

[149] Liberman U, Feldman MW. 1989 The reduction principle for genetic modifiers of the migration rate. In *Mathematical evolutionary theory* (ed. MW Feldman), pp. 111–137. Princeton, NJ: Princeton University Press.

[150] Altenberg L, Liberman U, Feldman MW. 2017 Unified reduction principle for the evolution of mutation, migration, and recombination. Proc. Natl Acad. Sci. USA **114**, E2392-E2400. (10.1073/pnas.1619655114) 28265103 PMC5373362

[151] Charlesworth B. 1976 Recombination modification in a fluctuating environment. Genetics **83**, 181-195. (10.1093/genetics/83.1.181) 1269919 PMC1213499

[152] Sasaki A, Iwasa Y. 1987 Optimal recombination rate in fluctuating environments. Genetics **115**, 377-88. (10.1093/genetics/115.2.377) 3557117 PMC1203087

[153] Otto SP, Michalakis Y. 1998 The evolution of recombination in changing environments. Trends Ecol. Evol. **13**, 145-151. (10.1016/S0169-5347(97)01260-3) 21238235

[154] Leigh EG J. 1970 Natural selection and mutability. Am. Nat. **104**, 301-305.

[155] Ishii K, Matsuda H, Iwasa Y, Sasaki A. 1989 Evolutionarily stable mutation rate in a periodically changing environment. Genetics **121**, 163-174. (10.1093/genetics/121.1.163) 17246489 PMC1203599

[156] Lachmann M, Jablonka E. 1996 The inheritance of phenotypes: an adaptation to fluctuating environments. J. Theor. Biol. **181**, 1-9. (10.1006/jtbi.1996.0109) 8796186

[157] Gillespie JH. 1981 The role of migration in the genetic structure of populations in temporally and spatially varying environments. III. Migration modification. Am. Nat. **117**, 223-233. (10.1086/283703) 982331

[158] McPeek MA, Holt RD. 1992 The evolution of dispersal in spatially and temporally varying environments. Am. Nat. **140**, 1010-1027. (10.1086/285453)

[159] Blanquart F, Gandon S. 2011 Evolution of migration in a periodically changing environment. Am. Nat. **177**, 188-201. (10.1086/657953) 21460555

[160] Caswell H. 1983 Phenotypic plasticity in life-history traits: demographic effects and evolutionary consequences. Am. Zool. **23**, 35-46. (10.1093/icb/23.1.35)

[161] Via S, Lande R. 1985 Genotype-environment interaction and the evolution of phenotypic plasticity. Evolution **39**, 505-522. (10.2307/2408649) 28561964

[162] Gavrilets S, Scheiner SM. 1993 The genetics of phenotypic plasticity. V. Evolution of reaction norm shape. J. Evol. Biol. **6**, 31-48. (10.1046/j.1420-9101.1993.6010031.x)

[163] de Jong G. 1995 Phenotypic plasticity as a product of selection in a variable environment. Am. Nat. **145**, 493-512. (10.1086/285752) 19425981

[164] Slatkin M. 1974 Hedging one’s evolutionary bets. Nature **250**, 704-705. (10.1038/250704b0)

[165] Seger J, Brockmann HJ. 1987 What is bet-hedging? In *Oxford surveys in evolutionary biology, vol. 4* (eds PH Harvey, L Partridge), pp. 182–211. Oxford, UK: Oxford University Press.

[166] Kussell E, Leibler S. 2005 Phenotypic diversity, population growth, and information in fluctuating environments. Science **309**, 2075-2078. (10.1126/science.1114383) 16123265

[167] Salathé M, Van Cleve J, Feldman MW. 2009 Evolution of stochastic switching rates in asymmetric fitness landscapes. Genetics **182**, 1159-1164. 19474199 10.1534/genetics.109.103333 PMC2728856

[168] Liberman U, Van Cleve J, Feldman MW. 2011 On the evolution of mutation in changing environments: recombination and phenotypic switching. Genetics **187**, 837-51. (10.1534/genetics.110.123620) 21212229 PMC3063677

[169] Carja O, Liberman U, Feldman MW. 2014 Evolution in changing environments: modifiers of mutation, recombination, and migration. Proc. Natl Acad. Sci. USA **111**, 17 935-17 940. (10.1073/pnas.1417664111) PMC4273399 25427794

[170] Taylor PD. 1990 Allele-frequency change in a class-structured population. Am. Nat. **135**, 95-106. (10.1086/285034)

[171] Taylor PD, Frank SA. 1996 How to make a kin selection model. J. Theor. Biol. **180**, 27-37. (10.1006/jtbi.1996.0075) 8763356

[172] Rousset F, Billiard S. 2000 A theoretical basis for measures of kin selection in subdivided populations: finite populations and localized dispersal. J. Evol. Biol. **13**, 814-825. (10.1046/j.1420-9101.2000.00219.x)

[173] Rousset F. 2003 A minimal derivation of convergence stability measures. J. Theor. Biol. **221**, 665-668. (10.1006/jtbi.2003.3210) 12713948

[174] Nowak MA, Sasaki A, Taylor C, Fudenberg D. 2004 Emergence of cooperation and evolutionary stability in finite populations. Nature **428**, 646-650. (10.1038/nature02414) 15071593

[175] Lessard S. 2005 Long-term stability from fixation probabilities in finite populations: new perspectives for ESS theory. Theor. Popul. Biol. **68**, 19-27. (10.1016/j.tpb.2005.04.001) 16023912

[176] Luo S. 2014 A unifying framework reveals key properties of multilevel selection. J. Theor. Biol. **341**, 41-52. (10.1016/j.jtbi.2013.09.024) 24096098

[177] Luo S, Mattingly JC. 2017 Scaling limits of a model for selection at two scales. Nonlinearity **30**, 1682. (10.1088/1361-6544/aa5499) 28867875 PMC5580332

[178] Cooney DB. 2019 The replicator dynamics for multilevel selection in evolutionary games. J. Math. Biol. **79**, 101-154. (10.1007/s00285-019-01352-5) 30963211

[179] Cooney DB. 2020 Analysis of multilevel replicator dynamics for general two-strategy social dilemma. Bull. Math. Biol. **82**, 66. (10.1007/s11538-020-00742-x) 32474720

[180] Weissing FJ. 1991 Evolutionary stability and dynamic stability in a class of evolutionary normal form games. In *Game equilibrium models I: evolution and game dynamics* (ed. R Selten), pp. 29–97. Berlin, Germany: Springer.

[181] Friedman D. 1991 Evolutionary games in economics. Econometrica **59**, 637-666. (10.2307/2938222)

[182] Eshel I, Motro U. 1981 Kin selection and strong evolutionary stability of mutual help. Theor. Popul. Biol. **19**, 420-433. (10.1016/0040-5809(81)90029-0) 7256682

[183] Eshel I. 1983 Evolutionary and continuous stability. J. Theor. Biol. **103**, 99-111. (10.1016/0022-5193(83)90201-1) 9156083

[184] Taylor PD. 1989 Evolutionary stability in one-parameter models under weak selection. Theor. Popul. Biol. **36**, 125-143. (10.1016/0040-5809(89)90025-7)

[185] Christiansen FB. 1991 On conditions for evolutionary stability for a continuously varying character. Am. Nat. **138**, 37-50. (10.1086/285203)

[186] Eshel I, Motro U, Sansone E. 1997 Continuous stability and evolutionary convergence. J. Theor. Biol. **185**, 333-343. (10.1006/jtbi.1996.0312) 9156083

[187] Hofbauer J, Sigmund K. 1990 Adaptive dynamics and evolutionary stability. Appl. Math. Lett. **3**, 75-79. (10.1016/0893-9659(90)90051-C)

[188] Dieckmann U, Doebeli M, Metz JAJ, Tautz D. 2004 Adaptive speciation. *No. 3.* *Cambridge studies in adaptive dynamics*. Cambridge, UK: Cambridge University Press.

[189] Weissing FJ, Edelaar P, van Doorn GS. 2011 Adaptive speciation theory: a conceptual review. Behav. Ecol. Sociobiol. **65**, 461-480. (10.1007/s00265-010-1125-7) 21423338 PMC3038232

[190] Ferrière R, Legendre S. 2013 Eco-evolutionary feedbacks, adaptive dynamics and evolutionary rescue theory. Phil. Trans. R. Soc. B **368**, 20120081. (10.1098/rstb.2012.0081) 23209163 PMC3538448

[191] Lewontin RC. 1977 Caricature of Darwinism. Nature **266**, 283-284. (10.1038/266283a0)

[192] Hamilton WD. 1977 ‘The selfish gene’. Nature **267**, 102-102. (10.1038/267102a0)16073394

[193] Wu CI. 1983 The fate of autosomal modifiers of the sex-ratio trait in drosophila and other sex-linked meiotic drive systems. Theor. Popul. Biol. **24**, 107-120. (10.1016/0040-5809(83)90035-7) 6658686

[194] Taylor JE, Jaenike J. 2002 Sperm competition and the dynamics of X chromosome drive: stability and extinction. Genetics **160**, 1721-1731. (10.1093/genetics/160.4.1721) 11973324 PMC1462075

[195] Leimar O. 2009 Multidimensional convergence stability. Evol. Ecol. Res. **11**, 191-208.

[196] Mullon C, Keller L, Lehmann L. 2016 Evolutionary stability of jointly evolving traits in subdivided populations. Am. Nat. **188**, 175-195. (10.1086/686900) 27420783

[197] Mullon C, Lehmann L. 2019 An evolutionary quantitative genetics model for phenotypic (co)variances under limited dispersal, with an application to socially synergistic traits. Evolution **73**, 1695-1728. (10.1111/evo.13803) 31325322

[198] Lande R. 1979 Quantitative genetic analysis of multivariate evolution, applied to brain:body size allometry. Evolution **33**, 402-416. (10.2307/2407380) 28568194

[199] Lande R, Arnold SJ. 1983 The measurement of selection on correlated characters. Evolution **37**, 1210-1226. (10.2307/2408842) 28556011

[200] Phillips PC, Arnold SJ. 1989 Visualizing multivariate selection. Evolution **43**, 1209-1222. (10.2307/2409357) 28564514

